# Longitudinal development of different dimensions of perfectionism in undergraduate medical students with respect to their medical school admission procedure

**DOI:** 10.3205/zma001252

**Published:** 2019-07-15

**Authors:** Daniela Vogel, Helen Seeliger, Sigrid Harendza

**Affiliations:** 1 University Medical Center Hamburg-Eppendorf, III. Department of Internal Medicine, Hamburg, Germany

**Keywords:** longitudinal development, medical school admission, perfectionism, personality traits, undergraduate medical education

## Abstract

**Objective: **The concept of perfectionism comprises high standards of performance as needed in medicine, but also concerns about making mistakes and dealing with social reactions about not being perfect. Perfectionism is associated with motivation and deep learning strategies but high expression of perfectionism has been found to be associated with symptoms of stress and anxiety in students. We aim to gain insights into the longitudinal development of different dimensions of perfectionism in medical students with respect to their way of medical school admission.

**Methods: **At the Medical Faculty of Hamburg University, 167 undergraduate medical students completed validated questionnaires (MPS-H and MPS-F) of different dimensions of perfectionism and sociodemographic data including medical school admission procedures, personality traits (BSI-10 and GSE), and symptoms of depression and anxiety (PHQ-9 and GAD-7) at the start of their first year and at half term of their second year.

**Results: **On average, after controlling for baseline and age, a significant decrease (p≤0.05) in Self-Oriented Perfectionism was found during the first two years in students who were admitted after a waiting period (M: -12.57; 95% CI: [-21.94 – -3.35]), by other ways of medical school entrance (M: -6.36; 95% CI: [-12.71 – -0.02]), by multiple mini-interviews (HAM-Int) (M: -5.52; 95% CI: [-9.90 – -1.14]), and by a natural science test (HAM-Nat) (M: -3.41; 95% CI: [-6.71 – -0.11]. Waiting period students also showed a significant longitudinal decline in the scale Personal Standards (M: -4.62; 95% CI: [-8.04 – -1.21].

**Conclusions: **Since medical students from all admission groups except from the high school degree group showed a significant longitudinal decrease in Self-Oriented Perfectionism, high levels of aspects of perfectionism associated with intrinsic motivation or deep learning strategies could be included medical school admission processes. Additionally, particular attention needs to be paid not to induce a loss of intrinsic motivation or deep learning strategies during undergraduate medical education.

## Zusammenfassung

**Zielsetzung: **Das Perfektionismuskonzept umfasst hohe Performanzstandards, wie sie in der Medizin erforderlich sind, aber auch Fehlersensibilität und das Umgehen mit sozialen Reaktionen auf nicht perfektes Verhalten. Perfektionismus steht in Zusammenhang mit Motivation und tiefen Lernstrategien, aber eine hohe Perfektionismusausprägung wurde bei Studierenden mit Stresssymptomen und Angstzuständen assoziiert gefunden. Ziel unserer Studie war es, Erkenntnisse über die longitudinale Entwicklung verschiedener Perfektionismusdimensionen bei Medizinstudierenden in Abhängigkeit ihrer Studienzulassung zu gewinnen.

**Methoden: **An der Medizinischen Fakultät der Universität Hamburg füllten 167 Medizinstudierende am Anfang ihres ersten und in der Mitte ihres zweiten Studienjahres validierte Fragebögen (MPS-H und MPS-F) zu verschiedenen Perfektionismusdimensionen aus und machten Angaben zu soziodemografischen Daten, die auch die Art der Studienzulassung, Persönlichkeitsmerkmale (BSI-10 und GSE) und Depressions- und Angstsymptome (PHQ-9 und GAD-7) umfassten.

**Ergebnisse:** Durchschnittlich fand sich nach Kontrolle für die Ausgangswerte und das Alter eine signifikante Abnahme (p≤0,05) in Selbstorientiertem Perfektionismus innerhalb der zwei Jahre für Studierende, die über Wartesemester (M: -12,57; 95% KI: [-21,94 – -3,35]), über andere Wege (M: -6,36; 95% KI: [-12,71 – -0,02]), über multiple Mini-Interviews (HAM-Int) (M: -5,52; 95% KI: [-9,90 – -1,14]) und über einen naturwissenschaftlichen Test (HAM-Nat) (M: -3,41; 95% KI: [-6,71 - -0,11] zum Medizinstudium zugelassen wurden. Wartesemester-Studierende zeigten darüber hinaus longitudinal eine signifikante Abnahme in Hohen Standards (M: -4,62; 95% KI: [-8,04 – -1,21].

**Schlussfolgerungen: **Da alle Studierenden bis auf die, die über die Abiturnote zugelassen wurden, eine signifikante longitudinale Abnahme in Selbstorientiertem Perfektionismus zeigten, könnten hohe Ausprägungen von Perfektionismusaspekten, die mit intrinsischer Motivation oder tiefen Lernstrategien verbunden sind, mit in den Zulassungsprozess einbezogen werden. Darüber hinaus sollte besonderes Augenmerk darauf gelegt werden, einen Verlust an intrinsischer Motivation oder an tiefen Lernstrategien während des Medizinstudiums nicht zu induzieren.

## Introduction

Medical students start their undergraduate studies at a young age. Being educated in a medical culture that emphasizes perfectionism, denial of personal vulnerability and delayed gratification can have deleterious effects on the well-being of physicians and lead to less-than-optimal care and medical errors [1]. It has been reported that medical students’ attitude scores decline with their progress through medical school, presumably due to their high attitude scores at entry and a loss of idealism during undergraduate training [2]. Such developments need to be detected as early as possible during undergraduate medical education and medical schools might wish to select students who will be able to keep the balance between working as perfect as possible without becoming seriously distressed. We found significant differences with respect to certain aspects of perfectionism in medical students who gained entry to medical school via different admission processes [3]. It has been shown that high scores for perfectionism are associated with psychiatric levels of distress in medical students [4] and that medical students show, for example, higher values in the perfectionism scale Personal Standards compared to arts students [5]. Perfectionism in general constitutes a multidimensional concept, which includes high standards of performance, concerns about making mistakes, and the social reaction and consequences about not being perfect [6], [7]. 

Students who were selected for undergraduate medical education by their high school degree showed high scores for Adaptive Perfectionism, which is associated with intrinsic motivation [3]. Maladaptive Perfectionism, which relates strongly to standards set externally, was the strongest predictor for symptoms of depression in newly admitted medical students [3]. Medical educators should be able to select the type of students who will be able to keep the balance between being motivated and working as perfect as possible without becoming seriously distressed. Therefore, measuring the expression of perfectionism at medical school entrance level stratified to the different admission procedures might be an additional aspect to select the desired applicants. However, certain aspects of perfectionism might change during undergraduate training but no data are available on the longitudinal development of perfectionism in medical students during their undergraduate medical training. 

In Germany, medical undergraduate studies comprise six years of training including two pre-clinical years, three clinical years and a final practice year. About 60% of medical school applicants in Germany can be selected by the universities themselves [https://www.hrk.de/fileadmin/redaktion/A4/Hochschulrahmengesetz__HRK_.pdf accessed 18.03.2019]. The most common selection test in Germany is the German Aptitude Test for Medical Studies [8] and other criteria, e.g. nursing practice [9], are used as well. The medical curriculum at the Medical Faculty of Hamburg University has been changed to a vertically integrated undergraduate model curriculum, which connects theoretical pre-clinical knowledge with clinical practice from the beginning [10]. Admission to this undergraduate medial program can be achieved in different ways:

1. by high school degree,
2. by a natural sciences test (HAM-Nat) [11],
3. by multiple mini-interviews (HAM-Int) [12],
4. after a waiting period, and
5. by other means, e.g. non-EU applicants or individuals from the military.

To date, aspects of perfectionism do not play a role in medical school selection. However, a longitudinal study shows an association between the scales Personal Standards, Concerns over Mistakes, and fear of negative social reactions with academic achievement and academic efficacy [13]. While academic achievements and expression levels in the scale Personal Standards influence each other bidirectionally in a positive way, academic efficacy predicted increases in the scale Personal Standards (perfectionistic strivings) and in academic achievement [13]. Furthermore, certain types of perfectionism, especially Socially-Oriented Perfectionism (SOP) have been shown to be significantly positively correlated with university students’ motivation and with adaptive learning strategies [14]. We were able to identify that medical students who were selected by different admission procedures show differences in the expression of certain dimensions of perfectionism at the beginning of the first year [3]. Therefore, the aim of this follow-up study was to gain insights into the development of different dimensions of perfectionism during the longitudinal course of the first two years at medical school. We also wished to study whether differences in the longitudinal development of perfectionism could be detected with respect to the different ways of medical school admission. 

## Methods

### Participants 

In October 2016, 358 students started their medical studies at the Medical Faculty of the University of Hamburg. They were asked to participate in our first study during the orientation week before the start of their first year. Students could participate if they had been enrolled for undergraduate medical studies at the University of Hamburg and if they participated in the orientation week and received the information about the study. A total of 298 students (189 female, 108 male) participated (response rate 83.2% at t1) [3]. All 358 students were invited again at midterm of their second year after a lecture to fill out the same questionnaire in January 2018. From these 358 students we received 215 questionnaires (response rate 60.1% at t2). Of these 215 students we could match 167 longitudinally (108 female students, 64%, 59 male students, 35%) who had also participated at t1 (response rate 77.6%). With respect to the originally participating 298 students the response rate was 56.0%. The mean age of these participants was 20.6 years at t1. With respect to the total cohort of 358 the longitudinal response rate was 46.6%. The distribution of participants in the different groups t1/t2 was as follows: High school degree: n=50/33; HAM-Nat: n=98/63; HAM-Int: n=66/34; Waiting period: n=51/21 and Others: n=33/15.

#### Data collection

The students responded again to questions about their sociodemographic data including an individualized code they had used at the beginning of the first year to identify the students’ questionnaires for assessment of their longitudinal development. The completion of the paper questionnaires took about 20 minutes. The Ethics Committee of the Hamburg Chamber of Physicians confirmed the innocuousness of this study and its congruence with the Declaration of Helsinki (WF-047/16). All questionnaires contained anonymized codes only decipherable by the students. 

#### Questionnaires

The instrument included again the German versions of the validated Multidimensional Perfectionism Scale by Hewitt and Flett (MPS-H) [7], [15], the Multidimensional Perfectionism Scale by Frost (MPS-F) [6], [16], the Big Five Inventory 10 (BFI-10) [17], the General Self-Efficacy Scale (GSE) [18], the Patient Health Questionnaire 9 (PHQ-9) [19], and the Generalized Anxiety Disorder 7 (GAD-7) [20]. The BFI-10, the GSE, PHQ-9, and the GAD-7 were selected in addition to sociodemographic data like age and sex to describe the admission groups [3]. Detailed descriptions about the instruments including their factors and scales are included in our previous study [3]. We also used the categories Adaptive and Maladaptive Perfectionism [5], which are composite measures of z-transformed scores of the MPS-H and the MPS-F. Adaptive Perfectionism (AP) is measured with the subscales Self-Oriented Perfectionism (SOP) and Personal Standards (PS) from the MPS-H and the MPS-F. Maladaptive Perfectionism is composed of the subscales Socially-Prescribed Perfectionism (SPP), Concern over Mistakes (CM) and Doubts about Action (DA) from the MPS-H and the MPS-F [5], [6], [7], [15]. Adaptive Perfectionism, appraised to be the healthier form of perfectionism, includes conscientiousness [21] and striving for high standards with feelings of accomplishment and satisfaction [22]. On the other hand, Maladaptive Perfectionism (MP) includes striving for high standards without feeling satisfied and paying much attention to the judgement by others, which is accompanied by higher levels of distress and neuroticism [5], [22]. Especially high levels of Socially-Prescribed Perfectionism (SPP), which are a characteristic of MP [5], were associated with emotional distress, anxiety, depression and lower academic achievement [23].

#### Statistical Analysis

Questionnaires were included in the statistical analysis, if at least 80% of the items of each scale were filled out per questionnaire in 2016 and in 2018. Missing data were replaced by the mean of the scale. If only one of the two item per a personality trait of the BFI-10 the scale was answered, we excluded this trait from the comparison. The data were analyzed using IBM SPSS statistics version 23 with an alpha level of .05. The difference of age was measured with a single analysis of variance with Sidak post hoc comparisons. For sex differences we used Chi-Quadrat. The Kruskall-Wallis-test was used for differences in the grade point average (GPA) with Dunn-Bonferroni-Tests as post hoc comparisons. To explore differences between the groups and the points of measurements, we conducted analyses of covariance (ANCOVA) with Sidak post hoc comparisons and η^2^ as effect size. To identify whether the way of medical school admission had an effect on the measured traits, we adjusted for t1 and calculated with the difference between t2 and t1 as dependent variable. We also used the age of the students at t1 (start of the first year) as additional covariate for the perfectionism traits because the admission groups showed a significant difference in this sociodemographic aspect and a correlation between age and perfectionism has been described [24]. Significant differences for the difference of measurements are reported by 95%-confidence intervals [95% CI]. Results are displayed with respect to students’ way of admission to medical school, i.e. grade point average of the high school degree, a natural sciences test (HAM-Nat), multiple mini interviews (HAM-Int), a waiting period or other means of admission. Means and standard deviations are reported as estimated means and confidence intervals. 

## Results

Table 1 (Tab. 1) shows the sociodemographic data of all students who longitudinally participated in the first (t1) and the second (t2) measurement differentiated by their way of medical school admission. Significant differences between the groups were found for age (F(4, 162)=123.17; *p*≤.001), sex (χ^2^(1, n=167)=16.57; *p*≤.005) and the grade point average of the high school degree (H(4)=104.93; *p*≤.001). No significant group differences were detected for personality traits, general self-efficacy, the patient health questionnaire, and generalized anxiety disorder. Longitudinally, students admitted by the HAM-Nat showed a significant (p=0.05) increase in openness, general self efficacy (GSE), and symptoms of depression (PHQ-9) (see table 2 (Tab. 2)). An increase (*p*≤0.05) in openness between the beginning of the first and mid-term of the second year was also found for the students who entered medical school by other means of admission (Others).

We detected one significant difference longitudinally within all groups of medical school admission except within students who were admitted by high school degree only and one additional significant difference within the group of students who were admitted after a waiting period (see table 3 (Tab. 3)). On average, we found a significant decrease in Self-Oriented Perfectionism during the first two years in students who were admitted after a waiting period (M: -12.57); 95% CI: [-21.79 – -3.35]), by other ways of medical school entrance (M: -6.36); 95% CI: [-12.71 – -0.02]) and by multiple mini-interviews (HAM-Int) (M: -5.52); 95% CI: [-9.90 – -1.14]) and the natural science test (HAM-Nat) (M: -3.41); 95% CI: [-6.71 – -0.11]. Furthermore, the 95% confidence interval of estimated means showed a significant longitudinal decline for the waiting period students in the dimension PS (Personal Standards) (M: -4.62); 95% CI: [-8.04 – -1.21].

Comparing the groups of medical school admission with respect to the differences in the perfectionism domains between t1 and t2, we did not find any significant differences when controlling for age. The highest explanation of variance for all admission groups, when controlling for age, can be shown for all perfectionism dimensions for the covariate of their baseline level (t1). For SOP, 8.9% of the variance (in total 11.3%) can be explained by its baseline level and for PS, 11.7% of the variance (in total 14.2%) can be attributed to its t1 scores. 

Table 4 (Tab. 4) shows the raw means of the perfectionism dimensions for the groups of medical school admission. An intriguing finding was that, while students from the high school admission group also showed a decrease between t1 and t2 in SOP (t1: 72.31±17.16 versus t2: 66.83±17.16) and PS (t1: 30.49±4.36 versus t2: 29.40±4.76), their results for these dimensions at t2 were still higher than the results for these dimensions for the students from the waiting period group at t1 (SOP, t1: 62.10±15.80; PS, t1: 26.10±4.31).

## Discussion

In this study, we longitudinally observed medical students’ development of different dimensions of perfectionism with respect to their medical school admission procedure. This was based on our previous observation of significant differences of perfectionism in students who were newly admitted to undergraduate medical studies by different procedures [3]. On average, four groups of students, HAM-Nat, HAM-Int, Waiting period, and Others, but not students who were only admitted by their high school grade showed a significant decrease in Self-Oriented Perfectionism (SOP) between their first two years of undergraduate medical studies. However, on the MPS-H perfectionism scale, medical students from all admission groups rated themselves at the beginning of the first year and still at mid-term of the second year as being highest in SOP compared to Other-Oriented Perfectionism (OOP) or Socially-Prescribed Perfectionism (SSP). SOP has been shown to have a significant positive correlation with university students’ motivation and with adaptive learning strategies [14]. According to this study, students with high SOP scores were motivated primarily by extrinsic compensation for their academic work and their SOP scores were also positively correlated with higher self-efficacy for learning and with intrinsic goal orientation for a specific task [14]. The longitudinal decrease of SOP in medical students in our study could be due to the socialization process at medical school with a possible decrease in intrinsic motivation due to high workload and frequent assessments [25], [26]. A positive correlation of intrinsic motivation and SOP has been demonstrated for undergraduate psychology students in their second semester [27]. The decrease of SOP is particularly worrisome for students who were admitted after a waiting period, because in addition to the steepest drop in their SOP score they showed a significant longitudinal decrease in the scale Personal Standards (PS). As SOP and PS are composite aspects of Adaptive Perfectionism, which is considered to be the healthier dimension of perfectionistic strivings [28], [29], they could comprise an interesting new focus in medical school selection procedures. The drop in PS may be at least partly due to students’ concepts of physicians’ daily practice and false expectations about the reality of undergraduate medical studies with a high work load, which is also a reason for students’ drop-out rates [30], which are highest for waiting period students [31]. 

Fabry and Giesler showed that medical students’ motivation [32] and their deep learning strategies [33] decrease significantly during the first year of undergraduate medical studies. This might be connected with or a consequence of the longitudinal decrease of medical students’ SOP we observed in our study, since SOP includes a salient motivation component [34]. Even though medical students selected by their high school degree showed a longitudinal, but not significant decrease in SOP, they still have a higher SOP score at mid-term of their second year than students who were selected after a waiting period showed at the time of medical school admission before the first year. Additionally, the students in our study who were admitted after a waiting period, were significantly older and had significantly lower high school grades than students from the other admission groups. Both aspects have been shown to be negatively correlated with academic performance in medical school and with higher dropout rates [31]. Since we found the lowest SOP score before the first year and the steepest decline in SOP at mid-term in the second year in the group of students who were admitted after a waiting period even when controlling for age, looking for adequately high SOP scores might be a useful additional criterion when selecting students for medical school. On the other hand, high scores for SOP in university students were also associated with higher worry and anxiety in exams [27]. This needs to be taken into account in undergraduate medical education and watched for by student mentoring and counselling when selecting students with high SOP scores.

In our study, we found a significant longitudinal increase in symptoms of depression in students who were admitted by the natural sciences test HAM-Nat. Furthermore, they showed an increase in Maladaptive Perfectionism, which was the strongest predictor for the occurrence of depression and anxiety symptoms in our previous study [3]. The students admitted to medical school by the HAM-Nat also showed the highest scores for Concerns over Mistakes (CM) and Doubts about Action (DA), which are the strongest predictors of depression [29], [35]. Additionally, the HAM-Nat students showed an increase in Socially Prescribed Perfectionism (SPP). High levels of SPP in university students were associated with greater test anxiety and a decreased likelihood of seeking help [14]. Furthermore, socially prescribed perfectionists were also more motivated by recognition from others rather than intrinsically [14]. Another longitudinal study showed a significant correlation in university students between high extrinsically motivated goal orientation and increasing levels of depression [36], which could be of relevance for our students selected by the HAM-Nat who might have been extrinsically motivated in their learning strategies to pass the natural sciences test. These students might also benefit from strategic counselling interventions and it would be important to detect their difficulties early enough to prevent their progress into symptoms of depression during their advancement in their undergraduate medical studies. Since learning factors and work environment were also reported as major drivers of medicals students’ and residents’ symptoms of burnout [37] longitudinal measurements of perfectionism levels during the advancement in undergraduate medical education might be useful in detecting students at risk for anxiety or depression.

Our study was performed at only one medical school, which will not allow generalizability of our findings. Furthermore, only 56% of the students who had participated at the beginning of their first year volunteered to fill out the questionnaires a second time at mid-term of their second year. This leads to further distortion of the proportion of students admitted via the different ways of medical school entry, which is not equal to begin with [3]. Additionally, the percentage of male and female students per admission group is different between the two points of data acquisition. There is also a difference of age and grade point average of the high school degree between the groups. Furthermore, BFI-10 and GSE, which were used as sociodemographic measures to characterize the groups did not play a role in the evaluation of perfectionism and could have been omitted. Despite these limitations, observation of different dimensions of perfectionism in undergraduate medical students through the course of their undergraduate curriculum might be worthwhile to observe important changes in certain dimensions of perfectionism, which might require counselling to prevent symptoms of depression or anxiety. Performing measurements of perfectionism might also be an interesting additional dimension in the processes of medical school admission to select students with high levels in the subscales Self-Oriented Perfectionism and Personal Standards who will be intrinsically motivated for their studies. In this case, the possible bias of socially desired answers has to be taken into account in the selection process. 

## Conclusions

The decrease in Self-Oriented Perfectionism (SOP) we longitudinally observed in medical students during their undergraduate education independent of medical school admission groups could be related to a decline in intrinsic motivation and deep learning strategies triggered by the demands of the curriculum. Further studies need to explore which factors are associated with this decline during undergraduate medical education. SOP, which includes intrinsic motivation, is highly relevant for physicians’ lifelong learning and work precision. Until factors for its longitudinal decrease during undergraduate medial training are known, medical school selection procedures, which admit students with high SOP values seem to be desirable. Measuring the level of perfectionism longitudinally during undergraduate medial training might provide an additional insight, which students will benefit from teaching about different learning strategies, mentoring or other means of counselling to prevent an increase in symptoms of depression. 

## Abbreviations

- AP: Adaptive Perfectionism
- BFI-10: Big Five Inventory 10
- CM: Concern over Mistakes
- DA: Doubts about Action
- GAD-7: Generalized Anxiety Disorder 7
- GSE: General Self-Efficacy Scale
- MP: Maladaptive Perfectionism
- MPS-H: Multidimensional Perfectionism Scale by Hewitt and Flett
- MPS-F: Multidimensional Perfectionism Scale by Frost
- PC: Parental Criticism
- PE: Parental Expectations
- PHQ-9: Patient Health Questionnaire 9
- PS: Personal Standards
- SOP: Self-Oriented Perfectionism
- SPP: Socially-Prescribed Perfectionism
- O: Organization
- OOP: Other-Oriented Perfectionism

## Acknowledgements

We would like to thank all students who participated in this study.

## Competing interests

The authors declare that they have no competing interests. 

## Figures and Tables

**Table 1:**
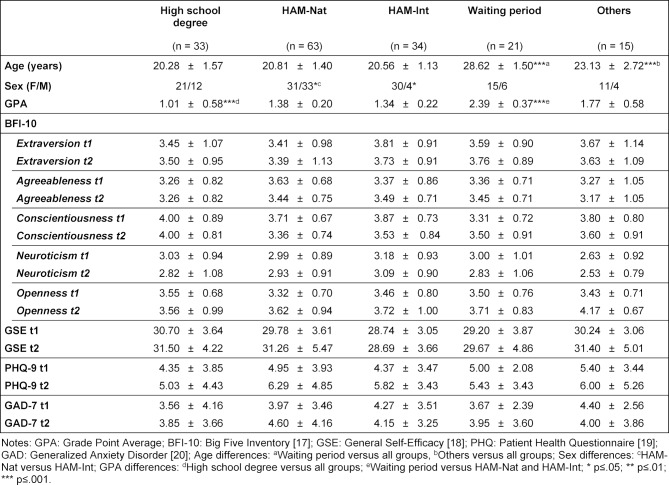
Sociodemographic data (age, sex, and grade point average) of the participants and longitudinal means and standard deviations of dimensions of personality, general self-efficacy, patient health questionnaire and generalized anxiety disorder

**Table 2:**
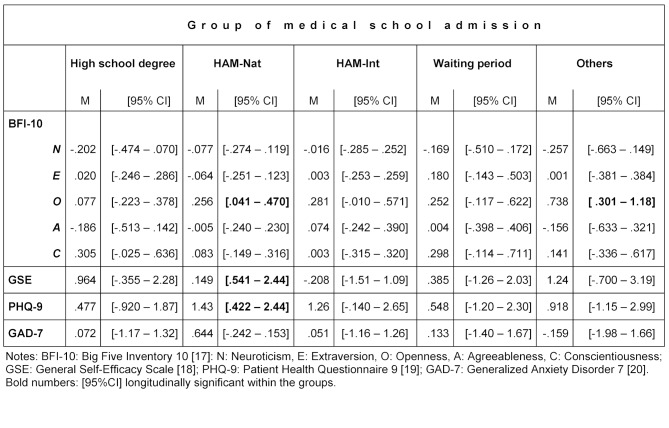
Estimated mean differences and confidence intervals in the dimensions of personality, self-efficacy, patient health and anxiety

**Table 3:**
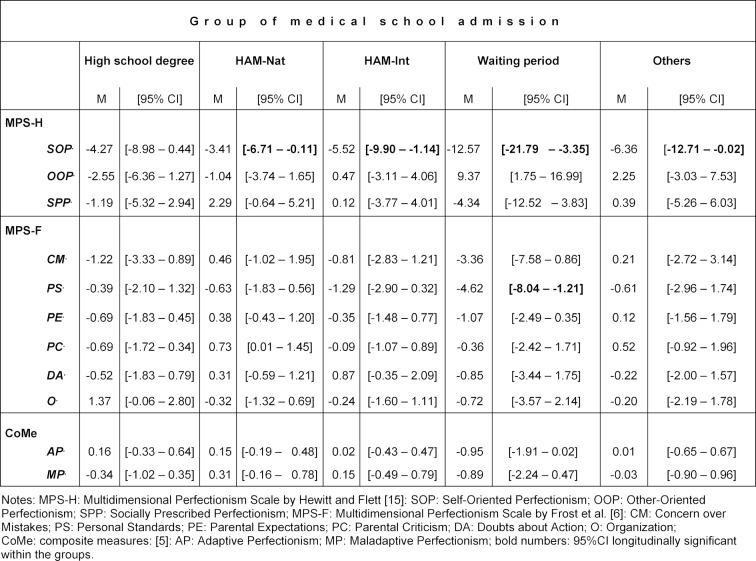
Estimated mean differences between t1 and t2 in the dimensions of perfectionism

**Table 4:**
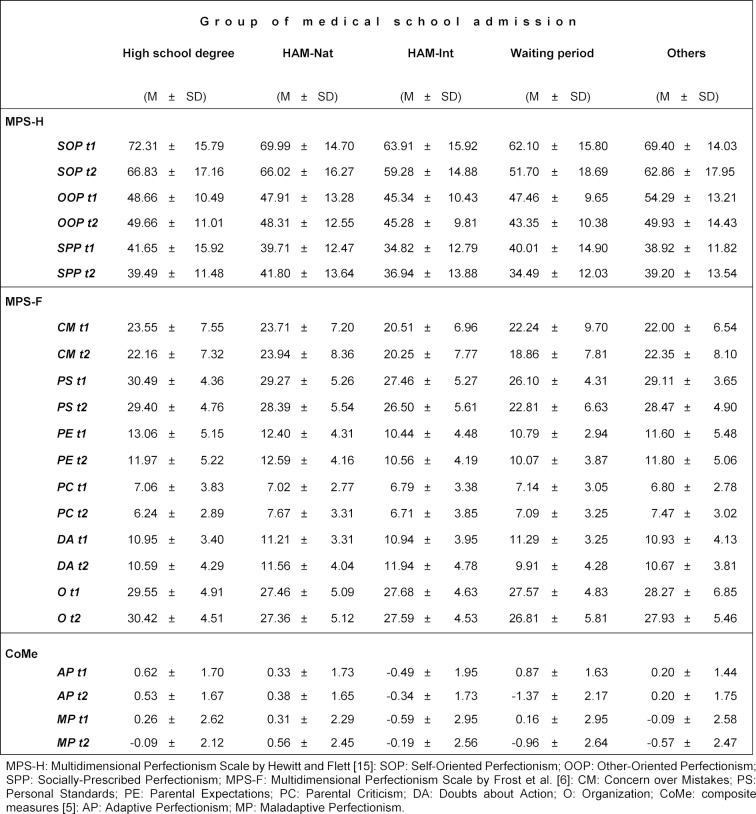
Means and standard deviations at t1 and t2 in the dimensions of perfectionism
